# Artificial intelligence-assisted measurements of coronary computed tomography angiography parameters such as stenosis, flow reserve, and fat attenuation for predicting major adverse cardiac events in patients with coronary arterial disease

**DOI:** 10.17305/bb.2024.10497

**Published:** 2024-10-01

**Authors:** Cheng Luo, Liang Mo, Zisan Zeng, Muliang Jiang, Bihong T Chen

**Affiliations:** 1 Department of Radiology, The First Affiliated Hospital of Guangxi Medical University, Nanning, China; 2 Department of Diagnostic Radiology, City of Hope National Medical Center, Duarte, California, USA

**Keywords:** Coronary computed tomography angiography (CCTA), artificial intelligence (AI), coronary artery disease (CAD), major adverse cardiac events (MACE)

## Abstract

Advancements in artificial intelligence (AI) offer promising tools for improving diagnostic accuracy and patient outcomes in cardiovascular medicine. This study explores the potential of AI-assisted measurements in enhancing the prediction of major adverse cardiac events (MACE) in patients with coronary artery disease (CAD). We conducted a retrospective cohort study involving patients diagnosed with CAD who underwent coronary computed tomography angiography (CCTA). Participants were classified into MACE and non-MACE groups based on their clinical outcomes. Clinical characteristics and AI-assisted measurements of CCTA parameters, including CT-derived fractional flow reserve (CT-FFR) and fat attenuation index (FAI), were collected. Both univariate and multivariable logistic regression analyses were performed to identify independent predictors of MACE, which were used to build predictive models. Statistical analyses revealed three independent predictors of MACE: severe stenosis, CT-FFR ≤ 0.8, and mean FAI (*P* < 0.05). Seven predictive models incorporating various combinations of these predictors were developed. The model combining all three predictors demonstrated superior performance, as evidenced by the receiver operating characteristic (ROC) curve, with an area under the curve (AUC) of 0.811 (95% confidence interval [CI] 0.774–0.847), a sensitivity of 0.776, and a specificity of 0.726. Our findings suggest that AI-assisted CCTA analysis, particularly using fractional flow reserve (FFR) and FAI, could significantly improve the prediction of MACE in patients with CAD, thereby potentially aiding clinical decision making.

## Introduction

Coronary artery disease (CAD) is a prevalent cardiovascular condition characterized by atherosclerotic lesions within coronary arteries, leading to stenosis and compromised blood flow, ultimately resulting in myocardial ischemia and cardiac events [1, 2]. Major adverse cardiac event (MACE) encompasses unstable angina, nonfatal myocardial infarction, and cardiac death [3]. Extensive research has explored various risk factors associated with MACE in patients with CAD, including gender, age, hypertension, and lipid profiles, to facilitate timely interventions and to enhance patient outcomes [4, 5].

Coronary computed tomography angiography (CCTA) is a valuable non-invasive imaging modality providing detailed information on coronary artery anatomy, plaque morphology, and luminal stenosis, enabling risk assessment and prognosis evaluation in patients with CAD [6–10]. While CCTA has proven its utility in assessing patients with moderate to severe CAD, advanced parameters, such as fractional flow reserve (FFR) and peri-coronary fat attenuation index (FAI) used to evaluate coronary hemodynamics and vascular inflammation have not been adequately assessed on conventional CCTA [11–14].

Artificial intelligence (AI) has gained increasing recognition in cardiac imaging for evaluating cardiac function and coronary arteries. AI’s integration into CCTA has propelled the capabilities in predicting and managing MACE in patients with CAD [15]. AI automates coronary artery segmentation, enhancing CCTA image interpretation, reducing processing times, and lessening reliance on expert radiologists [16]. It also aids in calculating the coronary artery calcium score (CACS), crucial for assessing disease risk by automating the identification and quantification of calcifications [16]. Additionally, AI improves the evaluation of coronary plaque characteristics and stenosis, FFR, and FAI, supporting early disease detection and helping prevent severe outcomes [17–21]. However, the application of AI in clinical settings, especially for measuring CCTA parameters predictive of MACE, is still emerging [22].

In this study, we used the AI algorithm to measure a spectrum of CCTA parameters, including coronary artery anatomy, FFR, and FAI, in a retrospective cohort of patients with CAD. Our objective was to identify risk factors and to build models for predicting MACE, potentially facilitating timely interventions, and improving outcomes for patients with CAD.

## Materials and methods

### Patient population

The retrospective study included patients diagnosed with CAD who exhibited moderate or severe stenosis in one or more of the three coronary artery trunks based on coronary CCTA findings [23]. Moderate stenosis was defined as luminal diameter stenosis equal to or exceeding 50% but less than 70%. In comparison, severe stenosis was defined as luminal diameter stenosis equal to or exceeding 70% but less than 90% [24]. This patient cohort was enrolled retrospectively at the First Affiliated Hospital of Guangxi Medical University, P.R. China, from January 2012 to December 2021.

**Figure 1.**
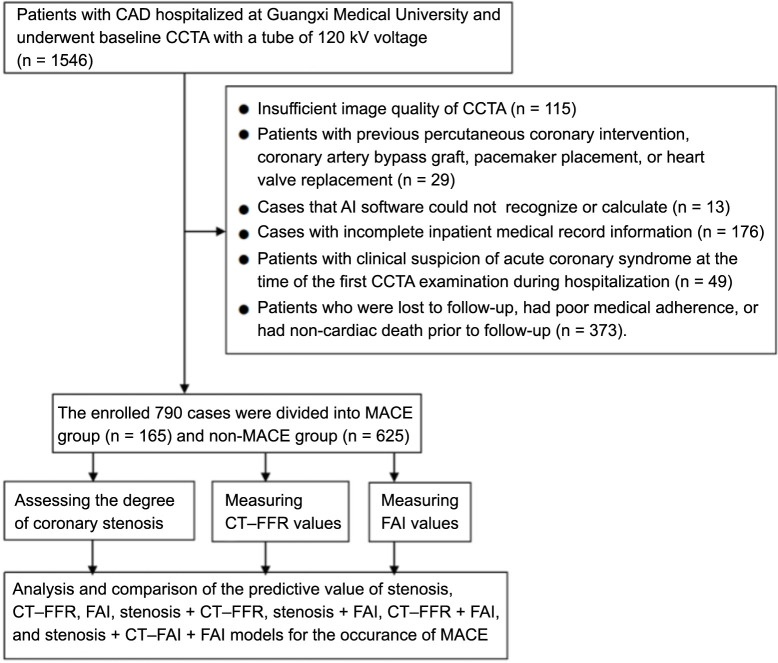
**Flow diagram of study cohort enrollment.** CAD: Coronary artery disease; CCTA: Coronary computed tomography angiography; AI: Artificial intelligence; MACE: Major adverse cardiac events; non-MACE: Without major cardiovascular events; CT-FFR: CT-derived fractional flow reserve; FAI: Fat attenuation index.

We focused on the patients with moderate and severe stenosis first because the patients with moderate and severe coronary arterial stenosis were more likely to be symptomatic due to myocardial ischemia and cardiac dysfunction [24]. This population of patients was more likely in need of urgent diagnosis and treatment for cardiac events as compared to patients with mild stenosis. This also made the AI-assisted CCTA parameter measurements with prompt data analysis and predictive modeling of potential cardiac events being more relevant to clinical scenarios. Second, by focusing and comparing patients with moderate and severe stenosis, more research could be performed to assess the impact of different degrees of stenosis on the prognosis of patients with CAD [25]. Exclusion criteria were set in the study design to ensure a sufficient cohort for data analysis. Patients with the following criteria were excluded: insufficient image quality, CCTA scans not processible by the AI software, previous coronary or cardiac interventions, incomplete medical records, acute coronary syndrome upon initial CCTA scan, loss to follow-up, poor medical adherence or non-cardiac death prior to follow-up. Patients were categorized into the MACE group and the non-MACE group based on the occurrence of MACE. The inclusion and exclusion criteria for this study are shown in Figure 1. Patient demographic and clinical data, including gender, age, height, weight, body mass index (BMI), smoking history, family history of CAD, and comorbidities such as diabetes, hyperlipidemia, and hypertension, were extracted from medical records (Table 1).

**Table 1:** Comparison of clinical data between the MACE and the non-MACE group

**Clinical data**	**MACE group (*n* ═ 165)**	**Non-MACE group (*n* ═ 625)**	***t*/χ^2^**	***P***
Age (years), mean ± SD	66.2 ± 11.0	66.1 ± 10.5	−0.229	0.819
Male, *n* (%)	128 (77.58)	399 (63.84)	11.090	0.001^*^
BMI (kg/m^2^), median (range)	24.49 (22.21–26.67)	24.28 (22.32–26.64)	−0.162	0.871
Smoking history, *n* (%)	63 (38.18)	231 (36.96)	0.083	0.773
Family history of CAD, *n* (%)	15 (9.09)	57 (9.12)	<0.001	0.991
Diabetes, *n* (%)	74 (44.85)	253 (40.48)	1.027	0.311
Hyperlipidemia, *n* (%)	38 (23.03)	156 (24.96)	0.262	0.608
Hypertensive disease, *n* (%)	129 (78.18)	485 (77.60)	0.026	0.873

*Indicates a statistically significant difference (*P* < 0.05). MACE: Major adverse cardiac events; non-MACE: Without major adverse cardiac events; BMI: Body mass index; CAD: Coronary artery disease; SD: Standard deviation.

All patients diagnosed with symptomatic CAD who underwent CCTA during the study period were eligible for inclusion. Patient follow-up data were collected through electronic medical record reviews and telephone interviews. Patients were monitored through electronic medical records and telephone follow-ups. The physician was not aware of the initial CCTA results during these follow-up visits. The primary endpoint was the occurrence of MACE. For patients who experienced MACE, the follow-up period was defined as the duration from the first CCTA examination to the onset of the first MACE. For those who did not experience MACE, the follow-up extended from the first CCTA examination to the date of the last telephone communication. In cases where multiple MACE occurred, the date of the initial MACE was considered the endpoint for follow-up [26]. MACE was identified when one or more of the following conditions were present: readmission due to unstable angina, with or without target vessel revascularization, nonfatal myocardial infarction, or cardiac death [3].

### Coronary computed tomography angiography (CCTA) imaging and analysis

CCTA images were acquired with five different CT scanners, including GE LightSpeed VCT (USA), Siemens SOMATOM Force (Germany), Siemens SOMATOM Definition Edge (Germany), Siemens SOMATOM Definition Flash (Germany), and GE Revolution CT (USA). A tube voltage of 120 kV was consistently employed for CT image acquisition to optimize the accuracy of FAI measurements [27].

**Figure 2.**
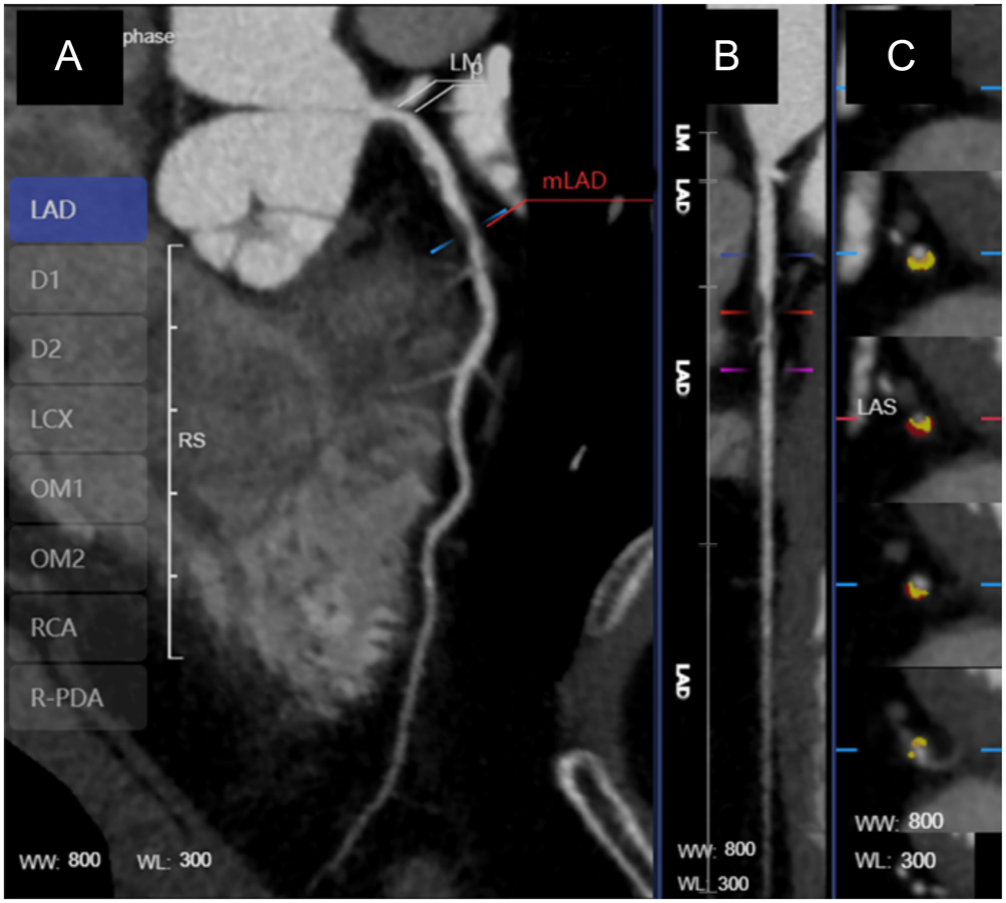
**Assessment of coronary artery stenosis on CCTA**. (A) Representative CCTA image showing the reconstructed bit images of coronary arteries; (B) Schematic diagram showing the measurement of the coronary stenosis represented by the luminal diameter stenosis rate as the ratio of the luminal diameter at the narrowest point to the luminal diameter of the normal vessels at both ends; (C) Color-coded rendering of the different components of a lesion plaque (yellow indicates the fiber component of the plaque, and red indicates the lipid component of the plaque). CCTA: Coronary computed tomography angiography; LAD: Left anterior descending branch; D1: First diagonal branch; D2: Second diagonal branch; LCx: Left circumflex; OM1: First obtuse marginal ramus; OM2: Second obtuse marginal ramus; RCA: Right coronary artery; R-PDA: Right posterior descending branch; LM: Left main coronary artery; mLAD: Middle section of left anterior descending branch.

The best time-phase images from the cardiac cycle in the CCTA dataset were selected and uploaded to the ShuKun Smart Medical Platform (Shukun (Beijing) Technology Co., Ltd., China) [28]. The AI models used for measuring CCTA parameters were built in the ShuKun Smart Medical Platform and the platform’s CoronaryDoc (version 5.0) software. The Shukun AI platform incorporated multiple deep learning compound networks. The technology featured optimal path detection capabilities, which allowed for the fully automatic segmentation and extraction of even third and fourth-level distal arterial vessels [28]. The platform’s CoronaryDoc (version 5.0) software was used for an initial assessment of coronary arteries. This assessment included determining the number of diseased vessels, identifying the vessel segment with the narrowest plaque, quantifying plaque volume, and evaluating the degree of stenosis (Figure 2). Plaques were categorized as high risk if they exhibited at least two of the following characteristics [29]: low attenuation plaque (LAP) with a central region attenuation density <30 Hounsfield units (Hu), positive remodeling (PR) with a ratio of the cross-sectional area of the vessel at the point of maximum stenosis to the average of the proximal and distal cross-sectional areas of the lesion being ≥ 1.1, napkin ring sign (NRS) characterized by a high-density halo around a low-density plaque (Hu ≤ 130), and spotty calcification (SC) denoting point-like calcification within the vessel wall < 3 mm in diameter and density >130 Hu, surrounded by non-calcified plaque.

**Figure 3.**
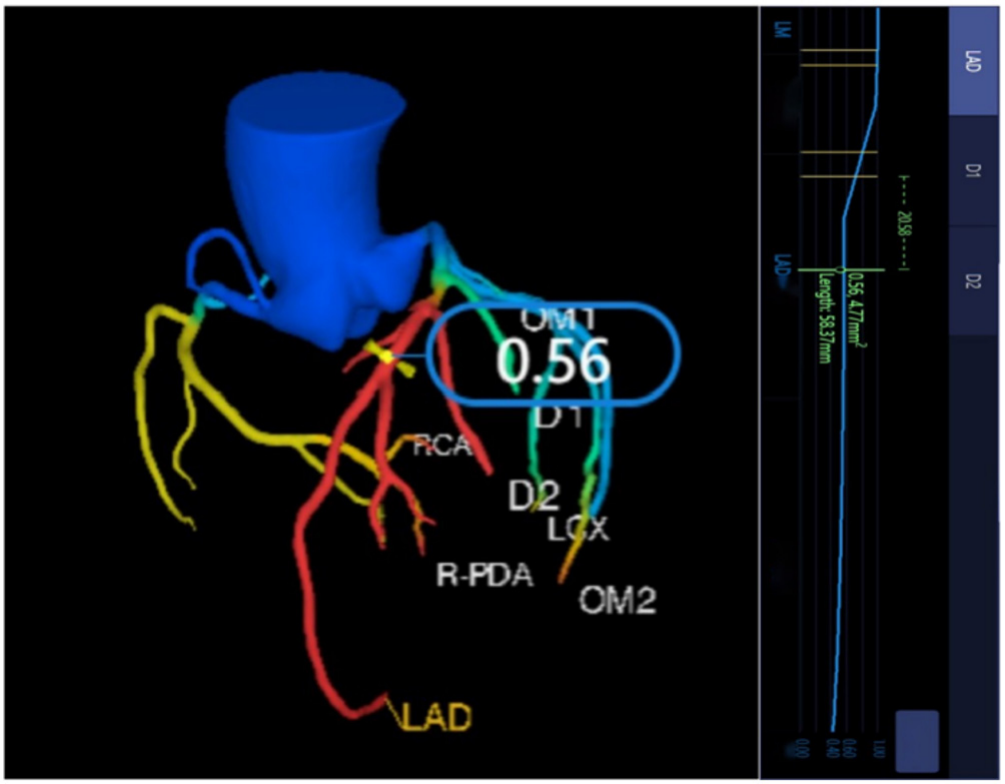
**Representative measurement of FFR values on CCTA using an AI software.** In the coronary dendrogram displayed on the AI software, the closer the vessel color is to blue, the higher the FFR value, the closer the vessel color is to red, the lower the FFR value. In this representative case, the FFR value is 0.56, measured at the narrowest part of the lesion plaque, about 2–3 cm from the vessel’s origin. FFR: Flow reserve fraction; CCTA: Coronary computed tomography angiography; AI: Artificial intelligence; LAD: Left anterior descending branch; D1: First diagonal branch; D2: Second diagonal branch; LCx: Left circumflex; OM1: First obtuse marginal ramus; OM2: Second obtuse marginal ramus; RCA: Right coronary artery; R-PDA: Right posterior descending branch; mLAD: Middle section of left anterior descending branch.

The platform’s Shukun-FFR (version 0.7) software was utilized to measure CT-derived FFR (CT-FFR) values [30]. The measurement location was manually selected at a point 2–3 cm distal to the narrowest part of the plaque (Figure 3) [31]. When CT-FFR values were ≤ 0.8, the case was considered positive and associated with a high risk for MACE [32].

**Figure 4.**
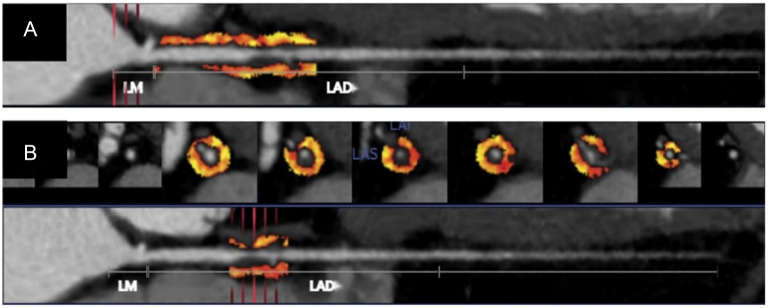
**Representative measurement of FAI values of the PCAT using an AI software.** (A) Automatic AI calculation of the FAI values around the three coronary arteries: The closer the color is to red, the higher the FAI value, the closer the color is to yellow, the lower the FAI value; (B) Automatic AI calculation of the FAI value and the plaque PCAT volume after manually selecting the lesion plaque range. FAI: Fat attenuation index; PCAT: Peri-coronary adipose tissue; AI: Artificial intelligence; LM: Left main coronary artery; LAD: Left anterior descending branch.

FAI values and the volume of peri-coronary adipose tissue (PCAT) were quantified using the platform’s Shukun-FAI (version 1.3) software [33]. Initially, the system automatically computed FAI values in the three primary coronary arteries: the right coronary artery (RCA), the left anterior descending (LAD) branch, and the left circumflex (LCx) branch (Figure 4A). Measurements for LAD and LCx extended from the beginning of the vessel to the distal 40 mm, while RCA measurements spanned from 10 mm to the distal 50 mm with a 4-mm extension to the outer vessel wall [24]. Using a similar approach, the average FAI value across the three main coronary arteries was calculated, along with FAI values specifically around lesioned plaques (Figure 4B). The measurement length corresponded to the plaque length, and the extension distance to the outer vessel wall matched the diameter of the diseased vessel. FAI measurements included voxels with attenuation values ranging from −190 to −30 Hu [24]. Additionally, the PCAT volume surrounding the lesioned plaque was automatically determined during FAI measurements.

The AI model in our study employed a deep learning architecture from the Shukun AI platform specifically designed for image analysis. Our AI model was trained using a dataset comprising CCTA images from a diverse cohort of patients diagnosed with CAD. This dataset was collected retrospectively from our hospital, including scans from January 2012 to December 2021. The dataset included over 790 CCTA scans reflecting a broad spectrum of patient demographics and disease severities. This diversity in the dataset helped in enhancing the model’s generalizability across different patient populations. The CCTA scans in the dataset were annotated by expert radiologists, providing a reliable ground truth for training the AI model. To prepare the data for training, all CCTA scans were re-sized and normalized to ensure uniformity in image quality and resolution, which was crucial for maintaining consistency in AI training.

### Predictive model development

There were a number of known risk factors for the occurrence of MACE in patients with CAD, such as age, male, BMI, smoking history, family history of CAD, diabetes, hyperlipidemia, and hypertensive disease and we compared these variables between the MACE group and the non-MACE group as presented in Table 1. In addition, we also analyzed and compared the CCTA imaging data between the two groups (Table 2). The significant variables from these analyses were subjected to univariate logistic regression analysis (Table 3). Subsequently, the significant variables from the univariate analysis were then included in the multivariate logistic regression analysis (Table 4). When there was a linear correlation between the significant variables, the analysis was conducted using the stepwise method in multivariate logistic regression analysis for the identification of independent predictors. Three predictive models were built using only one of the independent predictors. The remaining four models were built with all possible combinations of the independent predictors. The SPSS 25.0 software (IBM, Armonk, NY, USA) was used to perform receiver operating characteristic (ROC) curve analysis to obtain the area under the curve (AUC) value for assessing the predictive performance of each model for MACE. We used Medcalc 20.218 software (MedCalc Software Ltd., Belgium) to test the differences among the AUC values using the DeLong test. Statistical significance was set at *P* < 0.05.

**Table 2:** Comparison of CCTA imaging data between the MACE and the non-MACE group

**Image data**	**MACE group (*n* ═ 165)**	**Non-MACE group** **(*n* ═ 625)**	***t*/χ^2^**	***P***
Number of diseased vessels			9.796	0.007^*^
1, *n* (%)	63 (38.18)	318 (50.88)		
2, *n* (%)	69 (41.82)	225 (36.00)		
3, *n* (%)	33 (20.00)	82 (13.12)		
Distribution of major lesion vessel			4.393	0.111
RCA, *n* (%)	48 (29.09)	170 (27.20)		
LAD, *n* (%)	94 (56.97)	399 (63.84)		
LCx, *n* (%)	23 (13.94)	56 (8.96)		
Stenosis degree			61.659	<0.001^*^
Severe stenosis, *n* (%)	117 (70.91)	230 (26.80)		
Moderate stenosis, *n* (%)	48 (29.09)	395 (63.20)		
High-risk plaque, *n* (%)	68 (41.21)	167 (26.72)	13.119	<0.001^*^
LAP, *n* (%)	21 (12.73)	38 (6.08)		
PR, *n* (%)	93 (56.36)	338 (54.08)		
NRS, *n* (%)	25 (15.15)	73 (11.68)		
SC, *n* (%)	75 (45.45)	189 (30.24)		
The volume of major lesion plaque (mm^3^), median (range)	22.01 (8.92–43.09)	19.55 (7.96–44.85)	−0.466	0.641
PCAT volume of the main lesion plaque (mm^3^), median (range)	354 (200–668)	427 (273–664)	1.404	0.161
CT-FFR			96.966	<0.001^*^
CT-FFR ≤ 0.8, *n* (%)	140 (84.85)	261 (41.76)		
CT-FFR > 0.8, *n* (%)	25 (15.15)	364 (58.24)		
FAI around the major lesion plaque (Hu), mean ± SD	−72.44 ± 12.29	−78.23 ± 13.06	−5.127	<0.001^*^
RCA-FAI (Hu), mean ± SD	−72.37 ± 10.82	−78.46 ± 11.20	−6.258	<0.001^*^
LAD-FAI (Hu), mean ± SD	−73.70 ± 9.20	−78.64 ± 7.48	−7.180	<0.001^*^
LCx-FAI (Hu), mean ± SD	−70.49 ± 9.13	−74.63 ± 7.28	−6.129	<0.001^*^
Mean FAI (Hu), mean ± SD	−72.19 ± 8.64	−77.24 ± 6.89	−6.956	<0.001^*^

*Indicates a statistically significant difference (*P* < 0.05). CCTA: Coronary computed tomography angiography; MACE: Major adverse cardiac events; non-MACE: Without major adverse cardiac events; RCA: Right coronary artery; LAD: Left anterior descending branch; LCx: Left circumflex; LAP: Low attenuation plaque; PR: Positive remodeling; NRS: Napkin ring sign; SC: Spotty calcification; PCAT: Pericoronary adipose tissue; CT-FFR: CT derived fractional flow reserve; FAI: Fat attenuation index: Hu: Hounsfield unit.

**Table 3:** Univariate logistic regression analysis of significant variables

**Characteristics**	**OR**	**95% CI**	***P***
Male	1.959	1.313–2.925	0.001^*^
The number of diseased vessels: 1	–	–	–
The number of diseased vessels: 2	1.548	1.057–2.268	0.025^*^
The number of diseased vessels: 3	2.031	1.249–3.303	0.004^*^
Severe stenosis	4.186	2.882–6.080	<0.001^*^
High-risk plaque	1.923	1.345–2.748	<0.001^*^
CT-FFR ≤ 0.8	7.810	4.959–12.301	<0.001^*^
FAI around the major lesion plaque (Hu)	1.037	1.021–1.053	<0.001^*^
RCA-FAI	1.057	1.037–1.077	<0.001^*^
LAD-FAI	1.085	1.059–1.111	<0.001^*^
LCx-FAI	1.072	1.048–1.098	<0.001^*^
Mean FAI	1.098	1.071–1.127	<0.001^*^

*Indicates a statistically significant difference (*P* < 0.05). CT-FFR: CT-derived fractional flow reserve; FAI: Fat attenuation index; RCA: Right coronary artery; LAD: Left anterior descending branch; LCx: Left circumflex; OR: Odds ratio.

**Table 4:** Multivariate logistic regression analysis

**Characteristics**	**OR**	**95% CI**	***P***
Severe stenosis	2.695	1.766–4.114	<0.001^*^
CT-FFR ≤ 0.8	5.186	3.177–8.466	<0.001^*^
Mean FAI	1.094	1.065–1.124	<0.001^*^

*Indicates a statistically significant difference (*P* < 0.05). CT-FFR: CT-derived fractional flow reserve; FAI: Fat attenuation index; OR: Odds ratio; CI: Confidence interval.

### Ethical statement

The ethical approval for this study was obtained from The Medical Ethics Committee of First Affiliated Hospital of Guangxi Medical University (IRB: 2023-E217-01).

### Statistical analysis

Statistical analyses were performed using SPSS 25.0 (IBM, Armonk, NY, USA) and Medcalc 20.218 software (MedCalc Software Ltd., Belgium). Normally distributed continuous variables were expressed as mean ± standard deviation, and differences between groups were assessed using two-sample *t*-tests. Non-normally distributed continuous variables were presented as a median and interquartile range, with between-group differences analyzed using the Mann–Whitney *U* test. Categorical variables were described using counts and percentages, and group comparisons were conducted using the χ^2^ test. Statistical significance was set at *P* < 0.05.

## Results

### Comparison of clinical data and CCTA imaging data between the MACE group and the non-MACE group

In the cohort of 790 patients enrolled into the study for analysis, 165 patients (20.9%) were in the MACE group, while 625 patients (79.1%) were in the non-MACE group. The details for the patients within the MACE group included 152 patients admitted for unstable angina, 4 for nonfatal myocardial infarction, and 9 for cardiac deaths. The median follow-up duration for the MACE group was 30 months, ranging from 7 to 121 months. In contrast, the non-MACE group had a median follow-up of 47 months, ranging from 13 to 131 months. A significant difference was observed in gender distribution, the MACE group had a significantly higher proportion of males than the non-MACE group (*P* < 0.001). However, other factors, such as age, BMI, smoking history, family history of cardiac events, and comorbidities did not show significant differences between the two groups (all *P* > 0.05) (Table 1).

The results from AI-enhanced CCTA imaging measurements are presented in Table 2. Several CCTA parameters exhibited statistically significant differences between the MACE and non-MACE groups (*P* < 0.05). However, some lesion-specific metrics such as the distribution of major lesion vessels and the volume of major lesion plaques were not significantly different between the two groups (all *P* > 0.05).

### Logistic regression analyses of the factors influencing MACE

Through univariate logistic regression, an array of factors was noted with significant differences between the two groups, such as male sex, number of diseased vessels, severe stenosis, high-risk plaque, CT-FFR ≤ 0.8, and FAI around the major lesion plaque (Tables 1 and 2). Table 3 summarized these factors alongside their odds ratios (OR) and 95% confidence intervals (CI). Upon progressing to multivariate logistic regression analysis, three variables were identified as key independent predictors for MACE: severe stenosis, CT-FFR ≤ 0.8, and mean FAI (Table 4).

### Predictive models and their performance assessment

**Figure 5.**
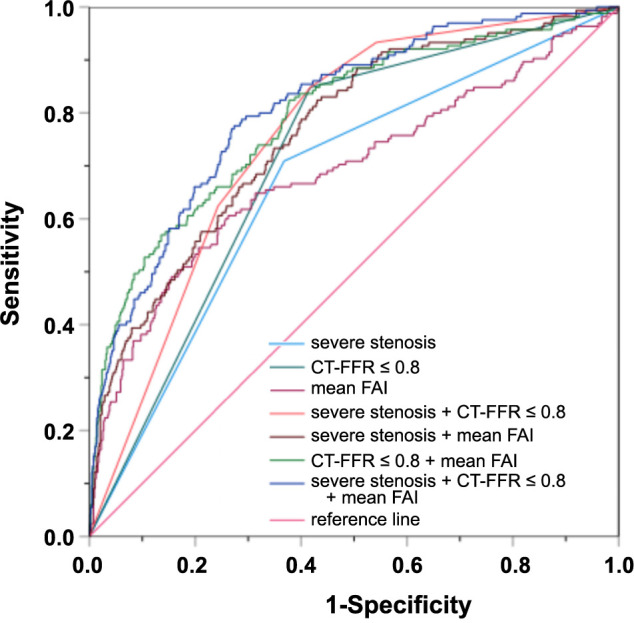
**ROC curves of models for predicting MACE.** ROC: Receiver operating characteristic; MACE: Major adverse cardiac events; CT-FFR: CT-derived fractional flow reserve; FAI: Fat attenuation index.

We constructed seven distinctive predictive models, leveraging the three predictors mentioned above in various combinations. Among these, the integrated model combining severe stenosis, CT-FFR ≤ 0.8, and mean FAI had the most robust performance. It attained an AUC of 0.811, a sensitivity of 0.776, and a specificity of 0.726 as presented in Figure 5 and Table 5.

**Table 5:** Data from receiver operating characteristic curve analysis of the models for predicting major adverse cardiac events

**Characteristics**	**Cut off value**	**AUC (95% CI)**	**Sensitivity**	**Specificity**	**PLR**	**NLR**
Severe stenosis		0.671 (0.625–0.716)	0.709	0.632	1.927	0.460
CT-FFR ≤ 0.8		0.715 (0.675–0.756)	0.848	0.582	2.029	0.261
Mean FAI	−73.24 Hu	0.689 (0.638–0.740)	0.606	0.738	2.313	0.534
Severe stenosis + CT-FFR ≤ 0.8		0.758 (0.720–0.795)	0.848	0.582	2.029	0.261
Severe stenosis + mean FAI		0.765 (0.724–0.805)	0.824	0.570	1.916	0.309
CT-FFR ≤ 0.8 + mean FAI		0.793 (0.752–0.833)	0.824	0.622	2.180	0.283
Severe stenosis + CT-FFR ≤ 0.8 + mean FAI		0.811 (0.774–0.847)	0.776	0.726	2.832	0.308

CT-FFR: CT-derived fractional flow reserve; FAI: Fat attenuation index; AUC: Area under the curve; CI: Confidence interval; PLR: Positive likelihood ratio; NLR: Negative likelihood ratio.

## Discussion

This study assessed the AI-assisted CCTA measurements for the assessment of independent risk factors for MACE in patients with CAD. We identified three independent predictors: severe stenosis, CT-FFR ≤ 0.8, and mean FAI. The predictive models built with all three predictors had robust performance in distinguishing the MACE group from the non-MACE group.

Our study results regarding the independent predictors for MACE were mostly consistent with the literature [4, 5, 34]. However, there were also divergent findings in our study when compared to the published studies. For instance, our study did not identify the high-risk plaque as a predictor of MACE, contrary to literature from pathological analysis of coronary plagues [35]. We speculated this might be due to our study using more stringent dual signs to determine high-risk plaques on CCTA images, which might have missed the less severe cases with high-risk plaques. In addition, the sign for “spotty calcification” was noted in a large percentage of lesions in our cohort, which might have affected the specificity of high-risk plaque and thus might have reduced its chances as a predictor of MACE.

Our study showing the CT-FFR ≤0.8 being an independent predictive risk factor for MACE was consistent with the published studies. Previous studies have shown the CT-FFR parameter being more efficient than the anatomic stenosis parameter for predicting MACE, and the CT-FFR ≤ 0.8 being associated with a higher incidence of the primary endpoint events, such as revascularization, acute myocardial infarction, and death [36–38]. However, prior studies have also shown the predictive power of CT-FFR being limited, and the accuracy of its predictive model being decreased in some instances, especially when the CT-FFR value being borderline between 0.7 and 0.8 [39]. Therefore, it is advisable to conduct a thorough evaluation of all relevant clinical and imaging data, utilizing the patients’ coronary arterial flow metrics to assess their risk of MACE.

Literature has shown the prognostic impact of fat around the coronary lesion plaque as represented by the FAI parameter from CCTA [40]. However, there was no agreement in the literature on the selection of a specific location in the coronary arteries for the measurement of FAI and for a cut-off value. We found the mean FAI around the three main coronary arteries, rather than the individual FAI value from each vessel, was the most significant independent risk factor at the cut-off value −73.24. In contrast, prior studies reported the LAD-FAI or the RCA-FAI as the most significant independent risk factors [24, 41]. The differences between our study and other’s data might be due to the variations in study design, differences in CCTA imaging protocols, our focus on patients with moderate to severe stenosis, and the use of AI-assisted measurements of MACE. Nevertheless, we speculated that the mean FAI around the three main coronary arteries might reflect multiple vessels, rather than a single vessel inflammation in the MACE group. Also, there might be a better reflection of the overall coronary arterial inflammatory status than the FAI value for an individual coronary vessel.

Our study also showed that the model integrating all independent predictors such as severe stenosis, CT-FFR ≤ 0.8, and mean FAI had the most robust performance to predict MAC as compared to the other models, which was consistent with the literature [42]. This observation supports the notion of using AI-assisted CCTA metrics in the comprehensive management of patients with CAD. AI has been increasingly used in cardiology [15, 43, 44]. Recent studies have emphasized the role of AI-driven algorithms in improving the accuracy of functional assessments in patients with CAD. Notably, AI could facilitate the identification of hemodynamically significant lesions, a crucial factor in managing patients with CAD [45, 46]. AI might also play a role in the assessment of patients’ overall cardiac health with both functional interpretation and anatomical depiction of coronary vessels on the CCTA images [16, 47]. The AI approach might help to delineate diagnostic pathways, optimize treatment planning, and improve patient outcomes.

This study contributed novel data to CAD research through an innovative AI approach. First, an integrated AI platform, as indicated in this study, should allow for rapid and accurate analysis of CCTA. This AI approach should help bridge the gaps between community-level hospitals with limited skills in imaging analysis and academic research-oriented hospitals with high-level expertise in imaging analysis. Second, the multiparametric prediction model we identified in this study might potentially improve the accuracy of predicting the occurrence of MACE and assist in clinical decision making especially for patients who did not have a clear-cut clinical indication for intervention. Third, our study identified new independent risk predictors of mean FAI values, which was a novel finding, although it needs to be confirmed in a large sample with a prospective study design.

There were several limitations to this study. First, there were inherent limitations associated with the retrospective design of this study. Various confounding variables could not be controlled in this retrospective study such as patients’ clinical demographic features, age, sex, comorbidities, treatment strategies, and prognosis. In addition, CT scanner parameters and imaging protocols could not be standardized in a retrospective design. Furthermore, it was a single-center study limiting the generalizability of our study results. A future study with a prospective design, multicenter approach, and a large sample size may be helpful to control these variables and validate the results from this study. Second, this study was also limited by the potential for selection bias. For instance, patients with mild coronary stenosis were not included in the study, which might have affected the overall study results. In addition, this study included a cohort with strict inclusion and exclusion criteria, which might not reflect clinical practice. The varying clinical follow-up time in this study was also an issue as some were up to 10 years after the initial CCTA. The lack of a standardized follow-up time might have affected the outcome assessment. More studies with a sufficient sample size and statistical power should be performed with the follow-up intervals being taken into consideration such as dividing the cohort into an early follow-up sub-cohort and a late follow-up sub-cohort. Third, there were potential limitations for AI applications in CCTA measurements. For instance, the accuracy of AI measurements was largely dependent on high image quality. Issues such as motion artifacts, imaging noise, suboptimal timing of contrast enhancement, and variations in imaging protocols might affect AI performance. In cases with poor image quality, the AI algorithms might not be able to accurately identify and segment the coronary arteries. Therefore, preprocessing measures were performed to minimize the impact of image quality on the algorithm’s performance. In addition, AI algorithms might have inherent biases from various aspects, such as biased training data, limited model architecture, and inconsistent parameter settings. For instance, if the training data was biased, containing only patients of specific age groups or with one type of coronary arterial stenosis, the algorithm might produce inaccurate predictions when processing other types of coronary arterial stenosis data. Lastly, AI algorithms were limited by interpretability as they were not intuitively understandable. This hindered the trust of the medical community and prevented wider clinical application of AI. Therefore, when applying AI to CCTA measurements, we incorporated methods to educate the referring physicians and to improve interpretability, such as using visual effects with charts and slide projections to present the learning and decision-making processes of the algorithm. Nevertheless, AI has advantages over manual assessment as it is more efficient, more accurate, more repeatable, and more adaptable for optimization and iteration of model algorithms.

## Conclusion

In this study, we identified independent risk factors and showed robust performance of our predictive models for cardiac events using AI-assisted CCTA metrics in patients with CAD. The non-invasive imaging focused AI approach may potentially help in clinical decision making and improve prognosis in patients with symptomatic CAD.

## Data Availability

All relevant data are within the paper.
